# Clinical documentation manual audit

**DOI:** 10.1186/1472-6963-11-S1-A20

**Published:** 2011-10-19

**Authors:** K Fan, M Wu, L Lau, K Lee, G Steve

**Affiliations:** 1 The Hong Kong Hospital Authority, Kowloon, Hong Kong

## Introduction

Preliminary audit studies in HA (Hospital Authority) hospitals have shown that diagnosis and procedure data reported in the electronic records were very accurate. However, appropriateness is as important as accuracy in clinical documentation.

With the introduction of internal resource allocation based on Casemix Pay for Performance (P4P), the relevance of clinical documentation became apparent. During the first year under the new P4P, there was significant improvement in clinical documentation, but large variations were observed between clusters in the extent to which specific clinical conditions were reported.

In addition, clinicians were puzzled by the perverse incentives to report diagnoses and procedures entirely for financial reasons. As a consequence, HA introduced the concept of grouping standards, that is, a series of agreed upon rules that would describe when specific International Classification of Diseases codes carry significant resource implications.

## Objective

During 2010-11, 22 grouping standards were developed through consultation with clusters and representatives of clinical specialties. In order to validate these standards, and to assess the accuracy and appropriateness of current documentation practices, a second and major manual audit was conducted.

## Methodology

This manual audit of approximately 10,000 patient records was undertaken in January and March 2011. A stratified, randomized sample of records was extracted, with approximately 30 records applied against each of the major hospitals. Each hospital’s records were audited using a predefined methodology by staff from other clusters or Hospital Authority Head Office.

## Results

This paper describes the manual audit and examines the implications of its results for appropriate clinical documentation.

## Conclusions

Auditing is an important tool in ascertaining the accuracy and appropriateness of clinical documentation practices, as well as in validating existing grouping standards.

